# Long-Term Air Pollution and Traffic Noise Exposures and Mild Cognitive Impairment in Older Adults: A Cross-Sectional Analysis of the Heinz Nixdorf Recall Study

**DOI:** 10.1289/ehp.1509824

**Published:** 2016-02-05

**Authors:** Lilian Tzivian, Martha Dlugaj, Angela Winkler, Gudrun Weinmayr, Frauke Hennig, Kateryna B. Fuks, Mohammad Vossoughi, Tamara Schikowski, Christian Weimar, Raimund Erbel, Karl-Heinz Jöckel, Susanne Moebus, Barbara Hoffmann, on behalf of the Heinz Nixdorf Recall study Investigative Group

**Affiliations:** 1 Environmental Epidemiology Group, IUF (Institut für umweltmedizinische Forschung)-Leibniz Research Institute for Environmental Medicine, Düsseldorf, Germany; 2 Department of Neurology, University Hospital of Essen, University of Duisburg-Essen, Essen, Germany; 3 Institute for Medical Informatics, Biometry and Epidemiology, University Duisburg-Essen, Essen, Germany; 4 Department of Chronic Disease Epidemiology, Swiss Tropical and Public Health Institute, Switzerland; 5 University of Basel, Basel, Switzerland; 6 Clinic of Cardiology, West German Heart Center, University Hospital of Essen, University Duisburg-Essen, Essen, Germany; 7 Deanery of Medicine, Medical Faculty, Heinrich Heine University of Düsseldorf, Düsseldorf, Germany

## Abstract

**Background:**

Mild cognitive impairment (MCI) describes the intermediate state between normal cognitive aging and dementia. Adverse effects of air pollution (AP) on cognitive functions have been proposed, but investigations of simultaneous exposure to noise are scarce.

**Objectives:**

We analyzed the cross-sectional associations of long-term exposure to AP and traffic noise with overall MCI and amnestic (aMCI) and nonamnestic (naMCI) MCI.

**Methods:**

At the second examination of the population-based Heinz Nixdorf Recall study, cognitive assessment was completed in 4,086 participants who were 50–80 years old. Of these, 592 participants were diagnosed as having MCI (aMCI, n = 309; naMCI, n = 283) according to previously published criteria using five neuropsychological subtests. We assessed long-term residential concentrations for size-fractioned particulate matter (PM) and nitrogen oxides with land use regression, and for traffic noise [weighted 24-hr (LDEN) and night-time (LNIGHT) means]. Logistic regression models adjusted for individual risk factors were calculated to estimate the association of environmental exposures with MCI in single- and two-exposure models.

**Results:**

Most air pollutants and traffic noise were associated with overall MCI and aMCI. For example, an interquartile range increase in PM2.5 and a 10 A-weighted decibel [dB(A)] increase in LDEN were associated with overall MCI as follows [odds ratio (95% confidence interval)]: 1.16 (1.05, 1.27) and 1.40 (1.03, 1.91), respectively, and with aMCI as follows: 1.22 (1.08, 1.38) and 1.53 (1.05, 2.24), respectively. In two-exposure models, AP and noise associations were attenuated [e.g., for aMCI, PM2.5 1.13 (0.98, 1.30) and LDEN 1.46 (1.11, 1.92)].

**Conclusions:**

Long-term exposures to air pollution and traffic noise were positively associated with MCI, mainly with the amnestic subtype.

**Citation:**

Tzivian L, Dlugaj M, Winkler A, Weinmayr G, Hennig F, Fuks KB, Vossoughi M, Schikowski T, Weimar C, Erbel R, Jöckel KH, Moebus S, Hoffmann B, on behalf of the Heinz Nixdorf Recall study Investigative Group. 2016. Long-term air pollution and traffic noise exposures and mild cognitive impairment in older adults: a cross-sectional analysis of the Heinz Nixdorf Recall Study. Environ Health Perspect 124:1361–1368; http://dx.doi.org/10.1289/ehp.1509824

## Introduction

Age-related cognitive decline is becoming increasingly important because of aging populations in developed countries. Since 1980, the prevalence of dementia has doubled each 5.5–6.7 years (Prince et al. 2013). The estimated prevalence of dementia will reach 42.7–48.1 million worldwide in 2020 (Prince et al. 2013). One way of characterizing the early stages of cognitive decline in elderly populations is mild cognitive impairment (MCI). MCI describes the stage between normal cognitive changes in aging and early dementia (Petersen et al. 1999). MCI can be classified as amnestic MCI (aMCI), where memory domains are affected and which most likely reflects the prodromal Alzheimer Disease (AD) stage, and nonamnestic MCI (naMCI), which has been linked to the prodromal stages of vascular and other forms of dementia (Petersen 2004).

Although a decline in cognitive functions is considered a normal consequence of aging (Glisky 2007), the identification of risk factors for dementia is of great importance for prevention and future treatment options. Several factors are related to dementia, such as age, ethnicity, sex, genetic factors, physical activity, smoking, drug use, education level, alcohol consumption, and body mass index (Chen et al. 2009). Approximately a decade ago, adverse effects of environmental exposures, such as air pollution, on the central nervous system were proposed (Oberdörster and Utell 2002). However, the effects of air pollution on the cognitive function of adults has not yet been thoroughly investigated (Block et al. 2012; Tzivian et al. 2015). The majority of studies investigating the effects of different pollutants on cognitive function are focused on childhood and adolescence (Guxens and Sunyer 2012). In adults, associations of air pollution with different aspects of cognitive function, mood disorders, and neurodegenerative diseases have been studied with partially inconsistent or even controversial results (Block et al. 2012). However, until now, most studies have generally supported the hypothesis that ambient air pollution is associated with cognitive function in long-term exposed persons (Tzivian et al. 2015).

An important inner-urban source of air pollution is traffic, which also emits ambient noise. Because of their common source, air pollution and traffic noise often occur simultaneously in time and space. Although air pollution and cognitive function have been studied repeatedly, the association of ambient noise with the cognitive function of adults has rarely been investigated (Clark and Stansfeld 2007; Tzivian et al. 2015). Most studies on ambient noise have examined short-term effects (Hygge et al. 2003; Schapkin et al. 2006; Stansfeld et al. 2000), suggesting a clinical impact of noise on psychological outcomes, for example, anxiety and annoyance. To our knowledge, there have been no long-term studies on the effects of traffic noise exposure on the cognitive function of adults. Furthermore, there are a limited number of studies that have investigated simultaneous co-exposures of air pollution and traffic noise on the cognitive function of adults.

The aim of this study was to investigate the independent cross-sectional associations of long-term exposure to air pollution and traffic noise in adults with diagnosed with MCI and its subtypes (amnestic and non-amnestic) using data from the first follow-up examination of the population-based Heinz Nixdorf Recall study in Germany.

## Materials and Methods

### Study Population

This study was a cross-sectional analysis based on data from the first follow-up examination (2006–2008) of the Heinz Nixdorf Recall (Risk factors, Evaluation of Coronary Calcium and Lifestyle) study, a population-based cohort study located in three adjacent cities (Bochum, Essen, and Mülheim/Ruhr) in the highly urbanized German Ruhr Area. The study design has been described in detail elsewhere (Schmermund et al. 2002). Briefly, 4,814 randomly chosen men and women who were 45–75 years old at baseline were enrolled into the study between December 2000 and August 2003. After 5 years (2006–2008), the first follow-up examination was performed (response rate of 90.2%). The Heinz Nixdorf Recall study was approved by the ethics committee of University Hospital Essen. All participants gave their written informed consent.

### Cognitive Assessment—MCI Diagnosis

At the 5 year follow-up examination, a cognitive performance assessment was implemented and completed for 4,086 participants. The cognitive performance assessment has been previously described in detail (Wege et al. 2011; Dlugaj et al. 2010). Briefly, it consists of established measures of immediate and delayed verbal memory (eight-word list, performance measured as number of words recalled in each trial), problem solving/speed of processing (labyrinth test, time in seconds needed to complete the task), verbal fluency (semantic category “animals,” number of recalled words within 1 min) and abstraction (as an executive function)/visual–spatial organization (clock-drawing test). The short cognitive performance assessment reached a good accuracy [area under the curve = 0.82, 95% confidence interval (CI): 0.78, 0.85]against a detailed neuropsychological and neurological examination assessing MCI in a previous study (Wege et al. 2011). The raw data for each subtest were *z*-transformed [mean = 0, standard deviation (SD) ± 1] according to three age groups (50–59 years, 60–69 years, and 70–80 years) and within every age group according to three education groups (≤ 10 years, 11–13 years, ≥ 14 years).

**Figure 1:**
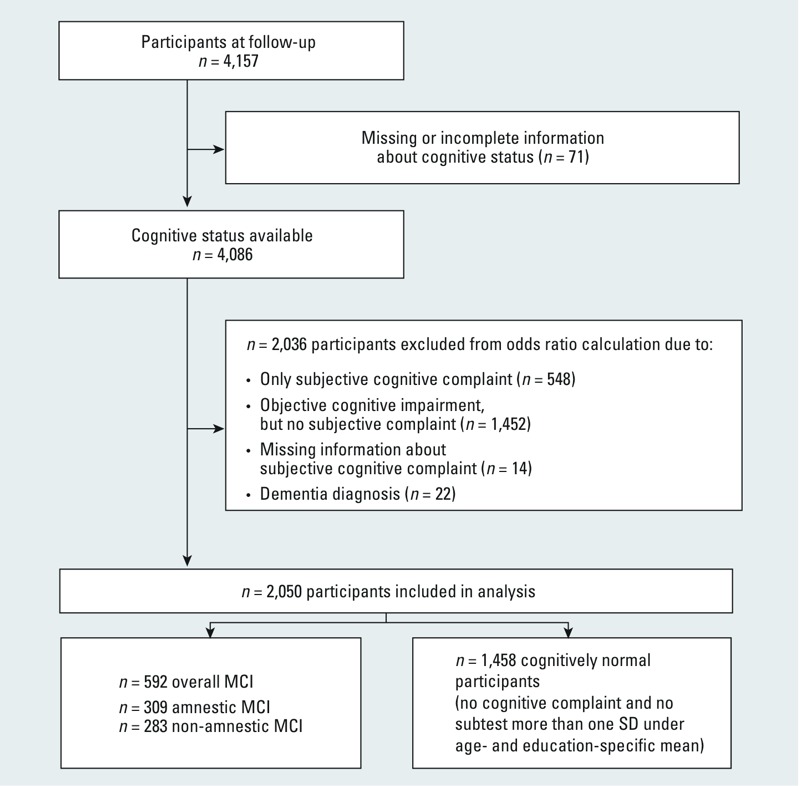
Derivation of the study population from participants of the Heinz Nixdorf Recall study.

MCI was diagnosed according to the Petersen/International Working Group on MCI criteria (Petersen 2004). Participants meeting the following criteria received an MCI diagnosis: *a*) presence of a subjective cognitive complaint (participants were asked if their cognitive performance had changed during the past 2 years. A complaint was considered present if the participant reported a decline in cognitive performance over time); *b*) presence of an objective cognitive impairment that was *c*) insufficient to fulfill criteria for dementia (Diagnostic and Statistical Manual of Mental Disorders, DSM-IV) and reflected *d*) generally intact activities of daily living. Presence of objective cognitive impairment (criterion *b*) was assessed using the results of all five cognitive subtests. Cognitive function was rated as impaired if the performance of at least one of cognitive subtests was more than one standard deviation (SD) below the age and education-specific mean (age- and education-specific *z*-scores), or if the participant received a score of ≥ 3 in the clock-drawing test. Participants with missing information on subjective cognitive complaints (*n* = 14) and participants who reported either a subjective cognitive complaint without objective cognitive impairment (*n* = 548) or who showed objective cognitive impairment without subjective cognitive complaint (*n* = 1,452) were excluded from the main analyses (Figure 1). We also excluded participants with a physician’s diagnosis of dementia or AD, with intake of cholinesterase inhibitors [Anatomic Therapeutic Chemical (ATC) classification code N06DA or other anti-dementia drugs (N06DX) as issued by the World Health Organization (WHO) (WHO 2004)], or who fulfilled the DSM-IV dementia diagnosis (and did not meet criterion 3 for Petersen MCI diagnosis) (*n* = 22).

Participants presenting an objective impairment in at least one memory domain (immediate and/or delayed verbal memory subtests) with or without impairment in any other cognitive domain received a diagnosis of amnestic MCI (aMCI) (Petersen 2004). If only nonmemory domains were impaired (at least one), the participant received a diagnosis of nonamnestic MCI (naMCI). Participants who presented neither a subjective cognitive complaint nor objective impairment were defined as “cognitively normal.”

### Exposure Assessment

We used the land-use regression model (LUR) according to the European Study of Cohorts for Air Pollution Effects (ESCAPE) standardized procedure (ESCAPE-LUR) (Beelen et al. 2007). Briefly, particulate matter of varying sizes with aerodynamic diameter measured in μm—less than 10 μm (PM_10_), > 2.5 to ≤ 10 μm (PMcoarse), less than 2.5 μm (PM_2.5_), and PM_2.5_ absorbance (blackness of the PM_2.5_-exposed filter, determined by measurement of light reflectance as a marker for soot and black carbon)—was measured at 20 sites, and nitrogen oxides (NOx and NO_2_) were measured at 40 sites in three separate 2 week periods (to cover different seasons) over 1 year (Beelen et al. 2013). Air pollution measurements were performed between October 2008 and October 2009, and the resulting LUR models were applied to estimate long-term exposure of concentrations at the baseline year of the study (Beelen et al. 2013; Eeftens et al. 2012). Background NO_2_ was modeled including the data from background measurement stations only while excluding traffic stations from the model (ESCAPE Project 2010). Annual averages (October 2008–September 2009) of measured pollutant concentrations at the monitoring sites and predictor variables, derived from Europe-wide and local geographic information system (GIS) databases, were used to develop the study-specific LUR model and to predict concentrations at each participant’s address. In the Ruhr Area, the models explained 88% of the variability in the annual concentrations of PM_2.5_, 77% of that for PM_10_, 66% of that for PMcoarse, 97% of that for PM_2.5_ absorbance, 84% of that for NO_2_, and 78% of that for NOx (Beelen et al. 2013; Eeftens et al. 2012).

Long-term exposure to traffic noise was modeled according to the European Directive 2002/49/EC (European Commission 2002) as the weighted 24-hr mean (L_DEN_) and the night-time (2200–0600 hours) mean (L_NIGHT_) at the baseline address, with consideration of the following determinants: small-scale topography of the area, dimensions of buildings, noise barriers, street axis, vehicle type–specific traffic density, speed limit, and type of street surface. Noise models were constructed for the cities, and traffic noise values were supplied as source-specific facade values from local city administrations. We used the most exposed facade values estimated at the residential addresses from the 2007 European noise exposure assessment (European Commission Working Group Assessment of Exposure to Noise 2007).

In addition to air pollution and noise exposure estimates, we used small-scale traffic indicators. The total traffic load at major roads (> 5,000 vehicles/day) in a 100-m buffer (vehicles × meters/day) was obtained from local road networks with traffic intensity data. Additional sensitivity analyses were performed using exposure variables from the European Air Pollution Dispersion and Chemistry Transport Model (EURAD-CTM) (Memmesheimer et al. 2004). This model used input data from official emission inventories on a spatial resolution of 1 km^2^ grid cells and included industrial sources, household heating, traffic and agriculture, and data on hourly meteorology and regional topography. Furthermore, pollutants entering the area by long-range transport were taken into account (Memmesheimer et al. 2004). The model output reflected the long-term urban background concentrations in the 1 km^2^ grid cell of the residential address of the participant. We used modeled averages of PM_2.5_, PM_10_, and NO_2_ for the years 2001–2003 to represent long-term exposure to air pollution.

### Covariates

Individual-level characteristics including age, sex, socioeconomic status [SES, assessed as education level, classified by the International Standard Classification of Education (ISCED) as total years of formal education, combining school and vocational training], alcohol consumption in drinks per week (one drink defined as 0.25 L beer, 0.1 L wine, or 0.02 L spirits), smoking status, environmental tobacco smoke (ETS, assessed as regular exposure to tobacco smoke at work, at home, or at other places), and any regular physical activity (regularly performing any type of sports activities) were assessed in standardized interviews and questionnaires. Anthropometry was measured according to standardized protocols, and body mass index (BMI) was calculated (kilograms per meters squared). Further intermediates included coronary heart disease (CHD), which was defined as a self-reported history of a myocardial infarction or coronary intervention at baseline or documented incidence of CHD during follow-up (Erbel et al. 2010); low-density lipoprotein (LDL)-cholesterol level measured using standard enzymatic methods; type 2 diabetes mellitus defined as fasting blood glucose greater than 125 mg/dL or blood glucose greater than 200 mg/dL or reported use of insulin or oral hypoglycemic agents within the last 7 days before examination; and use of statins and anti-hypertensive medication as categorized according to the Anatomical Therapeutic Chemical (ATC) classification index (WHO 2004) during the 7 days before examination. Apolipoprotein E (*APOE*) genotypes were investigated because the *APOE* ε4 allele has been shown to increase the risk for Alzheimer disease. Genotyping was performed using Cardio-Metabochip BeadArrays (Illumina, San Diego, CA, USA). Genotypes of two single-nucleotide polymorphisms (SNPs, rs7412 and rs429358) that distinguish between the three APOE alleles (ε2, ε3, and ε4) were extracted from the whole Metabochip data set. Genotyping was not available for 197 (4.86%) participants. Depressive symptoms were assessed using the German version of the Center for Epidemiologic Studies Depression scale (CES-D) short form (Hautzinger and Bailer 1993).

### Statistical Analysis

All air pollution components estimated with ESCAPE-LUR were obtained as continuous variables and included in the models per interquartile range (IQR). Noise exposure was investigated as a continuous variable with a threshold at 60 A-weighted decibels [dB(A)] for L_DEN_, and 55 dB(A) for L_NIGHT_, respectively, and calculated per 10 dB(A) increase. Threshold values were selected as those at which cardiovascular health effects have previously been seen (Babisch 2008). All noise values lower than the defined threshold value were equated to the threshold value. Total traffic load in major roads was adjusted for background NO_2_. Spearman correlation coefficients were calculated between estimated levels of air pollution and noise.

Multiple logistic regression models were constructed for each exposure. The main model included age, sex, SES (three categories: low, medium, and high according to ≤ 10, 11–13, and ≥ 14 years of education), alcohol consumption (categorized as 0, 1–3, > 3 and ≤ 6, > 6 drinks per week), smoking status (never, former, current), self-reported ETS (yes or no), any regular physical activity (yes or no), and BMI (continuous). To check potential nonlinear associations of age and BMI with MCI, we used quadratic and cubic polynomials, and the best model was chosen according to model fit using the adjusted *R* ^2^ criterion. In an extended analysis, the main model was adjusted for possible intermediate variables and potential risk factors: CHD diagnosis, LDL cholesterol level, intake of statin medications, diabetes mellitus, intake of anti-hypertensive medications, and city of residence. Additional adjustments of the main model were performed with APOE ε4 (carrier/non-carrier) and degree of depressive symptoms (continuous variable of CES-D score).

Two-exposure models for associations of noise and air pollution were developed to investigate the independent association of the two exposures.

### Effect Modification

We dichotomized air pollution concentrations at two cut points—one at the median and the second at the 75th percentile—and constructed product terms of air pollution (dichotomous) × noise. Noise variables were dichotomized on the threshold values [60 dB(A) for L_DEN_ and 55 dB(A) for L_NIGHT_] for interaction analysis with continuous air pollution variables. We also evaluated possible effect modification by age (< 65 vs. ≥ 65 years), sex, SES (low and medium vs. high education), BMI (≤ 30 vs. > 30), smoking status (non-smoker vs. current and former smoker), alcohol consumption (≤ 6 drinks per week vs. > 6 drinks per week), APOE ε4 (carrier vs. noncarrier), and depression (< 18 vs. ≥ 18 on the CES-D scale).

### Sensitivity Analysis

We performed sensitivity analyses for the main models excluding participants who changed their residential addresses between the baseline examination (2000–2003) and the first follow-up (2006–2008). Additionally, we performed a sensitivity analysis using the EURAD-CTM (Memmesheimer et al. 2004) air pollution model instead of the LUR exposure values.

We performed several sensitivity analyses to assess the degree of possible outcome misclassification. First, we added participants with objective impairment only to the group of participants classified as having overall MCI and added those with subjective complaints only to the cognitively healthy group. Second, we compared participants with overall MCI with all other participants combined, including participants with objective impairment only and those with subjective impairment only, in addition to those classified as cognitively healthy.

For noise variables, we performed a sensitivity analysis using different threshold values [65 dB(A) for L_DEN_ and 50 dB(A) for L_NIGHT_] and with a continuous noise variable without a threshold (per IQR of exposure). We also analysed noise variables in 10 dB(A) categories [≥ 45 to < 55 dB(A); ≥ 55 to < 65 dB(A); ≥ 65 to < 75 dB(A); ≥ 75 dB(A)].

We considered a *p*-value of 5% as statistically significant. We used SAS version 9.2 (SAS Institute Inc., Cary, NC, USA) and R version 2.13.1 (R Core Team 2013) software for analysis and processing of all databases.

## Results

We included 1,458 cognitively normal participants and 592 participants with MCI in our analyses; of the latter group, 309 had aMCI, and 283 had naMCI (Figure 1). The mean age of all participants combined was 64 years (63 years for the unimpaired group and 66 years for those with overall MCI) (Table 1). Proportions of men and women were generally consistent among the different outcome groups (unimpaired, all MCI, aMCI, and naMCI), and the majority had medium education. Most did not consume alcohol (32–49%) or had > 6 drinks/week (25–39%), and most were never smokers or ex-smokers, with approximately one-quarter of all participants reporting exposure to environmental tobacco smoke (ETS). Medication for hypertension was used by 45% of the unimpaired participants and 55% of those with MCI, and statin use was reported by 18% and 24%, respectively (Table 1).

**Table 1:** Main characteristics of the whole study population and its subgroups by outcome.

Variable/subgroups	Total population, *n *= 2,050	Unimpaired group, *n *= 1,458	Overall MCI, *n *= 592	Amnestic MCI, *n *= 309	Non-amnestic MCI, *n *= 283
Age (years), mean ± SD	64.1 ± 7.7	63.2 ± 7.4	66.3 ± 7.9	66.0 ± 8.0	66.6 ± 7.7
Men, *n* (%)	1,007 (49.1)	718 (49.2)	289 (48.8)	169 (54.7)	120 (42.4)
Education level, *n* (%)
Low	191 (9.3)	122 (8.4)	69 (11.7)	38 (12.3)	31 (10.9)
Medium	1,142 (55.7)	785 (53.8)	357 (60.3)	184 (59.5)	173 (61.1)
High	716 (34.9)	551 (37.8)	165 (27.9)	86 (27.8)	79 (27.9)
Alcohol consumption, *n* (%)
Never	726 (35.4)	468 (32.1)	258 (43.6)	152 (49.2)	106 (37.5)
1–3 drinks/week	400 (19.5)	284 (19.5)	116 (19.6)	55 (17.8)	61 (21.5)
> 3, ≤ 6 drinks/week	151 (7.4)	117 (8.0)	34 (5.7)	15 (4.8)	19 (6.7)
> 6 drinks/week	738 (36.0)	570 (39.1)	168 (28.4)	77 (24.9)	91 (32.2)
Smoking, *n* (%)
Current	462 (22.5)	327 (22.4)	135 (22.8)	75 (24.3)	60 (21.2)
Former smokers	720 (35.1)	520 (35.7)	200 (33.8)	112 (36.2)	88 (31.1)
Never smokers	868 (42.3)	611 (41.9)	257 (43.4)	122 (39.5)	135 (47.7)
Environmental tobacco smoke, *n* (%)	521 (25.4)	380 (26.1)	141 (23.8)	76 (24.6)	65 (23.0)
Any regular physical activity, *n* (%)	1,182 (57.7)	891 (61.1)	291 (49.2)	132 (42.7)	159 (56.2)
BMI (kg/m^2^), mean ± SD	28.1 ± 4.8	28.0 ± 4.6	28.4 ± 5.2	28.7 ± 5.3	28.0 ± 5.1
Diabetes, *n* (%)	369 (18.0)	238 (16.3)	131 (22.1)	74 (23.9)	57 (20.1)
CHD, *n* (%)	106 (5.2)	58 (4.0)	48 (8.1)	29 (9.4)	19 (6.7)
Medicated hypertension, *n* (%)	986 (48.1)	659 (45.2)	327 (55.2)	182 (58.9)	145 (51.2)
Medications – statins, *n* (%)	405 (19.8)	264 (18.1)	141 (23.8)	78 (25.2)	63 (22.3)
Cholesterol (mg/dL), mean ± SD	224.5 ± 40.8	225.0 ± 39.5	223.2 ± 43.9	222.6 ± 46.4	223.8 ± 41.1
Depression (CES-D score), mean ± SD	8.0 ± 6.6	6.6 ± 5.6	11.5 ± 7.6	12.0 ± 8.0	11.0 ± 7.1
*APOE*-ε4, *n* (%)	505 (24.6)	337 (23.1)	168 (28.4)	91 (29.4)	77 (27.2)
City, *n *(%)
Essen	650 (31.7)	443 (30.8)	207 (35.0)	93 (30.1)	97 (34.3)
Bochum	584 (28.5)	427 (29.3)	157 (26.5)	90 (29.1)	64 (22.6)
Mülheim	742 (36.2)	536 (36.8)	206 (34.8)	93 (30.1)	113 (39.9)
Abbreviations: BMI, body mass index; CES-D, Center for Epidemiologic Studies Depression scale; CHD, coronary heart disease; *APOE*, apolipoprotein E.					

The mean concentrations of PM_2.5_ and PM_10_ were 18.4 μg/m^3^ and 27.7 μg/m^3^, respectively (Table 2). Air pollution variables (ESCAPE-LUR) and noise variables correlated moderately (Spearman correlation coefficient: *r_s_ =* 0.30–0.48) (see Table S1).

**Table 2:** Descriptive statistics of exposure variables.

Exposure variables	Minimum	25th percentile	Median	75th percentile	Maximum	Mean ± SD
Air pollution variables
PM_2.5_ (μg/m^3^)	16.04	17.65	18.29	19.08	21.45	18.39 ± 1.05
PM_2.5_ absorbance (10^–5^/m)	1.01	1.37	1.52	1.72	3.39	1.58 ± 0.35
PMcoarse (μg/m^3^)	0.84	9.29	10.14	11.13	15.00	10.13 ± 1.53
PM_10_ (μg/m^3^)	23.97	26.54	27.43	28.62	34.68	27.74 ± 1.84
NO_2_ (μg/m^3^)	19.81	26.79	29.47	32.90	62.44	30.12 ± 4.85
NOx (μg/m^3^)	24.30	41.97	49.28	57.66	126.63	50.47 ± 11.70
Traffic load at major roads (vehicles × m/day) per 100,000	0.00	0.00	0.00	13.50	268.19	9.54 ± 21.20
Noise variables^*a*^
L_DEN_ [dB(A)]	0.00	46.70	52.13	60.87	84.56	53.74 ± 9.49
L_NIGHT_ [dB(A)]	0.00	38.15	43.54	51.75	76.29	44.88 ± 9.17
Abbreviations: dB(A), A-weighted decibels; L_DEN_, weighted 24-hr mean; L_NIGHT_, night-time mean. ^***a***^Descriptive statistics for the noise exposures are based on continuous variables, without a threshold.						

### Associations between Air Pollution, Noise and MCI

We found positive associations of most exposures with overall MCI and aMCI (Table 3). For example, an IQR increase in PM_2.5_ and PM_2.5_ absorbance and a 10 dB(A) increase in L_DEN_ was significantly associated with overall MCI with odds ratios (OR) of 1.16 (95% CI: 1.05, 1.27), 1.11 (95% CI: 1.03, 1.19), and 1.40 (95% CI: 1.03, 1.91), respectively, in the main model. For aMCI, these associations were slightly stronger, with ORs of 1.22 (95% CI: 1.08, 1.38), 1.17 (95% CI: 1.03, 1.35), and 1.53 (95% CI: 1.05, 2.24), respectively. Associations of MCI and its subtypes with other investigated air pollutants were similar to but slightly lower than associations with PM_2.5_. Associations of L_NIGHT_ with MCI and its subtypes were slightly higher than those obtained with L_DEN_ (Table 3). All AP and noise exposures were more strongly associated with aMCI than with overall MCI or naMCI. Traffic indicator variables were not associated with MCI or its subtypes.

**Table 3:** Associations of air pollution and noise with MCI*^a^*, OR (95% CI).

Cognitive criterion	PM_10_ (IQR = 2.09 μg/m^3^)	PMcoarse (IQR = 1.00 μg/m^3^)	PM_2.5_ (IQR = 1.44 μg/m^3^)	PM_2.5_ absorbance (IQR = 0.35 × 10^–5^/m)	NO_2 _(IQR = 6.11 μg/m^3^)	NOx (IQR = 15.70 μg/m^3^)	Traffic load at major roads (vehicles × m/day)^*b*^	L_DEN_ [threshold 60 dB(A)]	L_NIGHT_ [threshold 55 dB(A)]
Overall MCI	1.11 (0.99, 1.23)	1.11 (0.98, 1.26)	1.16 (1.05, 1.27)	1.11 (1.03, 1.19)	1.10 (0.97, 1.25)	1.10 (0.96, 1.26)	1.00 (0.94, 1.07)	1.40 (1.03, 1.91)	1.80 (1.07, 3.04)
Amnestic MCI	1.17 (1.07, 1.35)	1.26 (0.95, 1.33)	1.22 (1.08, 1.38)	1.17 (1.03, 1.35)	1.13 (1.01, 1.38)	1.13 (0.96, 1.34)	1.03 (0.96, 1.11)	1.53 (1.05, 2.24)	2.25 (1.23, 4.12)
Nonamnestic MCI	1.04 (0.90, 1.21)	1.09 (0.92, 1.29)	1.10 (0.92, 1.31)	1.03 (0.90, 1.19)	1.01 (0.85, 1.20)	1.05 (0.88, 1.26)	0.95 (0.85, 1.05)	1.26 (0.82, 1.93)	1.31 (0.60, 2.85)
Abbreviations: dB(A), A-weighted decibels; IQR, interquartile range; L_DEN_, weighted 24-hr mean; L_NIGHT_, night-time mean; MCI, mild cognitive impairment. ^***a***^Adjusted for age, sex, socioeconomic status, alcohol consumption, smoking status, self-reported environmental tobacco smoke, any regular physical activity, body mass index. ^***b***^Additionally adjusted for background NO_2_.									

**Figure 2:**
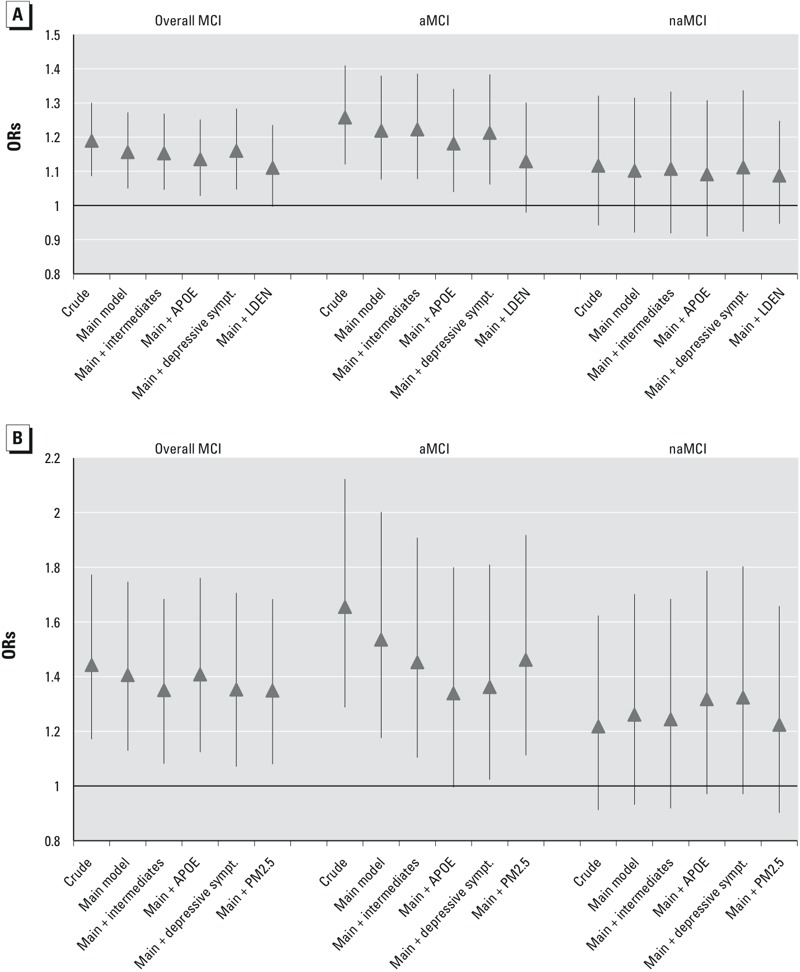
Associations between environmental exposures and overall mild cognitive impairment (MCI), amnestic MCI (aMCI), and nonamnestic MCI (naMCI) for crude, main and extended models. (*A*) Association of PM_2.5_ [per interquartile range (IQR)] with overall MCI, aMCI, naMCI. (*B*) Association of weighted 24-hr average (LDEN) {per 10 A-weighted decibels [dB(A)]} with overall MCI, aMCI, naMCI. Main model adjusted for age, sex, socioeconomic status, alcohol consumption, smoking status, self-reported environmental tobacco smoke, any regular physical activity, and body mass index. Covariates classified as “intermediates” were coronary heart disease diagnosis, low-density lipoprotein cholesterol level, diabetes mellitus and intake of statin or anti-hypertensive medication.

Point estimates for associations with PM_2.5_ and L_DEN_ were robust to different model specifications (Figure 2). Results for associations with other air pollutants and with L_NIGHT_ were also robust to adjustment (data not shown). Additional adjustment of the main model with potential intermediate variables (CHD diagnosis, LDL cholesterol level, diabetes mellitus, and intake of statin or anti-hypertensive medication) did not change the association of PM_2.5_ with MCI and its subtypes; however, these adjustments slightly attenuated the association of L_DEN_ with overall MCI and aMCI (Figure 2). Adjustment for *APOE* slightly decreased the association of PM_2.5_ with MCI and aMCI and the association of L_DEN_ with aMCI (Figure 2). Adjustment for depressive symptoms did not change the association between PM_2.5_ and MCI and its subtypes, but the point estimate for the association of L_DEN_ with aMCI decreased after adjustment for depressive symptoms (Figure 2).

Associations of PM_2.5_ with overall MCI and aMCI were positive but became nonsignificant after adjustment for noise (L_DEN_ or L_NIGHT_), whereas positive associations of L_DEN_ with MCI and aMCI remained significant after adjustment for PM_2.5_ (Figure 2). ORs for other air pollutants and overall MCI or aMCI also remained positive but were not significant when adjusted for noise (data not shown). For example, in the two-pollutant model for NO_2_ adjusted for L_DEN_, the association with aMCI was OR = 1.12 (95% CI: 0.97, 1.28).

### Effect Modification

Associations between PM_2.5_ and MCI were stronger in participants with no or moderate alcohol consumption [OR = 1.27 (95% CI: 1.07, 1.50)] than in participants with high alcohol consumption [OR = 0.96 (95% CI: 0.75, 1.21; p_inter_ = 0.05)] and in former and current smokers [OR = 1.39 (95% CI: 1.12, 1.71)] than in nonsmokers [OR = 1.01 (95% CI: 0.85, 1.21; p_inter_ = 0.02)] (see Figure S1). Other interactions were non-significant, but associations between PM_2.5_ and MCI were stronger in participants with high noise exposure [e.g., OR = 1.30 (95% CI: 1.01, 1.67) compared with OR = 1.10 (95% CI: 0.93, 1.29) for L_DEN_ ≥ 60 and < 60, respectively; p_inter_ = 0.28] and for participants with depressive symptoms [OR = 1.35 (95% CI: 0.89, 2.05)] compared with other participants [OR = 1.13 (95% CI: 0.97, 1.31); p_inter_ = 0.43]. For the association of L_DEN_ with MCI, we observed a tendency towards a higher susceptibility in carriers of the *APOE* risk allele [OR = 1.99 (95% CI: 1.11, 3.56)] compared with others [OR = 1.21 (95% CI: 0.83, 1.78); p_inter_ = 0.17] and in participants with high PM_2.5_ exposure [OR = 1.53 (95% CI: 1.17, 2.00)] compared with those exposed to low PM_2.5_ [OR = 1.08 (95% CI: 0.73, 1.62); p_inter_ = 0.17].

### Sensitivity Analysis

After excluding participants who changed their residential addresses between the baseline and the first follow-up examination, the group of participants with overall MCI contained 511 participants (86.3% of the whole MCI sample); of these, 259 had aMCI and 252 had naMCI. Restricting the sample to nonmovers did not change the effect estimates or the significance level of the observed associations.

Correlation between air pollution variables modeled using ESCAPE-LUR and EURAD-CTM was moderate to high (*r*_s_ = 0.44–0.77). PM_10_ modeled according to the EURAD-CTM was associated with naMCI [OR = 1.20 (95% CI: 0.98, 1.49) per IQR (4.19 μg/m^3^)] in the main model. However, PM_2.5_ modeled with the EURAD-CTM was not associated with MCI (data not shown).

In sensitivity analyses adding participants with only objective impairment to the MCI cases and in those adding participants with only subjective complaints to the cognitively healthy group, we observed slightly lower associations than those obtained in the main analysis, and the results partially became nonsignificant. For example, for the associations of PM_2.5_ and L_DEN_ with objective impairment, the ORs were 1.10 (95% CI: 1.01, 1.21) and 1.18 (95% CI: 0.98, 1.44), respectively. The results for participants with overall MCI versus all other participants (adding all unclear cases to the cognitively healthy group) were similar. For example, for the associations of PM_2.5_ and L_DEN_, we obtained ORs of 1.10 (95% CI: 0.97, 1.26) and 1.19 (95% CI: 0.91, 1.56), respectively.

The results of sensitivity analyses for noise variables with a 65-dB(A) threshold for L_DEN_ and a 50-dB(A) threshold for L_NIGHT_ and for continuous noise variables showed similar results to those of the main analysis (see Table S2). Categorical analysis of noise variables revealed elevated estimates > 65 dB(A) (see Table S3).

## Discussion

We found that long-term exposure to both air pollution and road traffic noise was associated with overall MCI, particularly with the amnestic subtype, in this middle- and older-aged German study population. In two-exposure models including both PM_2.5_ and L_DEN_, effect estimates for both exposures remained positive and the association with noise remained statistically significant for overall MCI and aMCI. Our results also indicated that the two investigated environmental exposures may interact with each other. Specifically, associations of PM_2.5_ with overall MCI were stronger among those exposed to higher levels of noise, and the association of L_DEN_ with overall MCI appeared to be limited to those with high exposure to PM_2.5_. However, differences between groups defined by high or low noise or PM_2.5_ were not significant.

The association between long-term exposure to air pollution and MCI confirms the findings of previous studies that have reported associations of different air pollutants with accelerated neurocognitive decline in longitudinal studies (Tonne et al. 2014; Weuve et al. 2012) and in cross-sectional studies (Chen and Schwartz 2009; Loop et al. 2013; Power et al. 2011). We also found that long-term exposure to traffic noise (both L_DEN_ and L_NIGHT_) was positively associated with MCI. Similar to the association with air pollutants, the association with ambient noise (both L_DEN_ and L_NIGHT_) was stronger for aMCI than for naMCI. This is a novel finding; studies investigating the association between ambient noise and cognitive functions in the general adult population are scarce (Wright et al. 2014; Basner et al. 2014). Importantly, our results showed that positive associations of environmental exposures with MCI continued to be evident when adjusted for confounding by the other exposure. If corroborated by other studies, this finding has important public health implications regarding protection of the public.

Previous studies on air pollution and subtypes of MCI or specific domains of neurocognitive function are scarce, and their results are inconsistent. In a cross-sectional study investigating associations between PM_2.5_, O_3_, and NO_2_ with attention, memory, and executive functions in 1,496 residents of Los Angeles, California (Gatto et al. 2014), and in a longitudinal study investigating the effects of PM_2.5_ and PM_10_ on the decline of inductive reasoning, verbal fluency, and verbal memory in 2,867 older residents of London, U.K. (Tonne et al. 2014), air pollution was associated with reduced verbal and logical memory, respectively, and in a cross-sectional analysis of NHANES data for 1,764 U.S. adults (Chen and Schwartz 2009), the association of PM_10_ with memory function disappeared after adjustment for personal covariates. In line with the findings reported by Gatto et al. (2014) and by Tonne et al. (2014), we found highly consistent associations of air pollution and traffic noise with memory-related aMCI. This outcome is potentially of great public health importance because aMCI may be associated with an elevated risk of developing AD (Petersen 2004). An association of air pollution with AD was previously reported in an animal study by Calderón-Garcidueñas et al. (2004).

The association between aMCI as a prodromal AD stage and air pollution seems plausible from a biological perspective. There is evidence for increased brain accumulation of beta-amyloid, a hallmark of AD, in dogs with high exposure to air pollution (Calderón-Garcidueñas et al. 2008). Furthermore, an experimental study of rats exposed to diesel exhaust by inhalation over 4 weeks or as a single intratracheal administration reported a link between air pollution and neuroinflammation (Levesque et al. 2011), which also plays an important role in the development of AD (Block and Calderón-Garcidueñas 2009). Additionally, in an animal study, Arnsten and Goldman-Rakic (1998) reported that in monkeys, mild noise exposure significantly impaired performance in spatial working memory, which is dependent on prefrontal cortex function, and elicited excessive dopamine release (Arnsten and Goldman-Rakic 1998). Because there is a lack of evidence regarding the mechanisms of long-term noise exposure, we can only speculate whether these mechanisms could also be responsible for long-term effects of noise on cognitive function.

We did not find a significant association between air pollution and naMCI, although the odds ratios were elevated. In contrast, in a longitudinal study by Kioumourtzoglou et al. (2016) that assessed the effects of PM_2.5_ on neurological hospital admissions among Medicare enrollees in the northeastern United States, city-wide long-term exposure to PM_2.5_ was associated with hospital admission for Parkinson disease, which is closely related to naMCI (Costello et al. 2011). In turn, naMCI is related to vascular dementia (Petersen 2004), which is strongly associated with cardiovascular disease (Paciaroni and Bogousslavsky 2013). Chronic exposure to air pollution has been linked to an elevated risk of cardiovascular disease (Brook et al. 2010), and our own previous study of the Heinz Nixdorf Recall study population found associations between long-term PM air pollution and risk factors for or manifestations of atherosclerosis and cardiovascular disease (Hoffmann et al. 2007), suggesting that one possible pathway from air pollution to naMCI and vascular dementia could be mediated via cardiovascular disease. However, we did not find strong evidence to support this pathway in the present analysis.

In general, we found the strongest associations for PM_2.5_ and less-clear associations for PM_10_. The particle fraction that might be responsible for potential effects on neurocognition is not clear. Although some studies have reported strong associations of cognitive function with smaller particles or with traffic-related exposures and soot (Loop et al. 2013; Ranft et al. 2009), others observed associations with larger particle fractions (Chen and Schwartz 2009). Only a few studies have comprehensively compared the associations between cognitive outcomes and different particle size fractions and air pollution components (Chen and Schwartz 2009; Weuve et al. 2012), and these studies have yielded different results. For example, in a cohort study by Weuve et al. (2012) that investigated the effects of PM_2.5_, PM_2.5–10_, and PM_10_ on global cognition, verbal memory, and executive function in 10,409 participants in a 7-year follow-up examination, an association of PM_2.5–10_, but not of PM_2.5_ or of PM_10_, with global cognitive decline was found, and in a study by Chen and Schwartz (2009), such an association was found only for PM_10_. Clearly, more combined toxicological and epidemiological research is needed to identify the most pathogenic components of air pollution and to enhance our understanding of the biology of adverse air pollution effects.

### Strengths and Limitations of the Study

This study was performed using a database of a middle- and older-age population in the highly urbanized German Ruhr area. Unless more studies with other study populations and methods are conducted in different areas of the world, the generalizability of the present findings cannot be assessed. One important limitation of this study is its cross-sectional design, which prevented us from establishing a temporal relationship between air pollution/noise and MCI. In addition, cognitively impaired people were probably less likely to have participated in the study, which could have led to selection bias. Another limitation of our study is the absence of detailed information on room location, type of windows, and other factors that can contribute to misclassification of both noise- and air-pollution exposure. Additionally, some of the personal variables (alcohol consumption, physical activity, smoking status) were obtained from questionnaires, which can lead to residual confounding in case of imprecision and underreporting. We also cannot exclude possible exposure misclassification and residual confounding between air pollution and noise exposures because they share a common source and are moderately correlated.

Our study has several strengths. To our knowledge, this is the first study that has investigated the association of different air pollutants and noise with cognitive function in two-exposure models. Additionally, this is the first study that has assessed the effects of air pollution and noise in participants with MCI. Because these participants have a higher risk of developing dementia, the longitudinal follow-up will allow us to examine the relationship between air pollution and cognitive decline. In our study, we investigated associations of air pollutants and noise with both clinically important MCI subtypes, aMCI and naMCI. Furthermore, we excluded all participants with either only objective impairment or only subjective cognitive complaints, resulting in a reference group of cognitively healthy participants. The large range of pollutants and the extensive adjustment for covariates in this extremely well-characterized population-based study sample enabled good control of confounding factors. The population-based nature of this study and the standardized outcome assessment methods, as well as the large sample size, are additional strengths.

## Conclusions

Long-term exposures to air pollution and traffic noise were both associated with MCI, particularly the amnestic subtype, in this middle- and older-age German study population. In two-exposure models including both PM_2.5_ and traffic noise, positive associations persisted for both exposures, and associations with noise remained statistically significant for overall MCI and aMCI.

## Supplemental Material

(505 KB) PDF
