# Supplementary material for: Long-Term Air Pollution and Traffic Noise Exposures and Mild Cognitive Impairment in Older Adults: A Cross-Sectional Analysis of the Heinz Nixdorf Recall Study
Source: Environ Health Perspect. 2016 Feb 5;124(9):1361–8. doi: 10.1289/ehp.1509824 (PMC5010410; doi:10.1289/ehp.1509824)
Supplement: (505 KB) PDF [file ehp.1509824.s001.acco.pdf]

Environ Health Perspect
DOI: 10.1289/ehp.1509824

Note to readers with disabilities: *EHP* strives to ensure that all journal content is accessible to all readers. However, some figures and Supplemental Material published in *EHP* articles may not conform to 508 standards due to the complexity of the information being presented. If you need assistance accessing journal content, please contact ehp508@niehs.nih.gov. Our staff will work with you to assess and meet your accessibility needs within 3 working days.

Supplemental Material

Long-Term Air Pollution and Traffic Noise Exposures and Mild Cognitive Impairment in Older Adults: A Cross-Sectional Analysis of the Heinz Nixdorf Recall Study

Lilian Tzivian, Martha Dlugaj, Angela Winkler, Gudrun Weinmayr, Frauke Hennig, Kateryna B. Fuks, Mohammad Vossoughi, Tamara Schikowski, Christian Weimar, Raimund Erbel, Karl-Heinz Jöckel, Susanne Moebus, and Barbara Hoffmann, on behalf of the Heinz Nixdorf Recall Study Investigative Group

Table of Contents

Table S1. Spearman correlations between air pollution (ESCAPE-LUR) and noise variables (r_s)
Table S2. Association of noise with MCI, OR (95% CI)
Table S3. Association between L_{DEN} (categorical) and overall MCI
Figure S1. Effect modification of association between exposures and overall MCI. Panel A: Associations of $PM_{2.5}$ (per IQR) with overall MCI. Panel B: Associations of L_{DEN} (per 10 dB(A)) with overall MCI. Main model adjusted for age, sex, SES, alcohol consumption, smoking status, self-reported ETS, any regular physical activity and BMI.

Table S1. Spearman correlations* between air pollution (ESCAPE-LUR) and noise variables (r_s)

**Variable**	**$PM_{2.5}$ (µg/m^3)**	**$PM_{2.5}$ absorbance (10^{-5}/m)**	**PMcoarse (µg/m^3)**	**PM_{10} (µg/m^3)**	**NO_2 (µg/m^3)**	**NOx (µg/m^3)**	**Traffic load at major roads (veh*m/d)**	**L_{DEN} (dB(A))**
$PM_{2.5}$ absorbance (10^{-5}/m)	0.89							
PMcoarse (µg/m^3)	0.69	0.74						
PM_{10} (µg/m^3)	0.88	0.89	0.70					
NO_2 (µg/m^3)	0.65	0.62	0.46	0.54				
NOx (µg/m^3)	0.63	0.52	0.42	0.51	0.93			
Traffic load at major roads (veh*m/d)	0.20	0.39	0.20	0.20	0.53	0.34		
L_{DEN} (dB(A))	0.30	0.48	0.32	0.31	0.37	0.29	0.59	
L_{NIGHT} (dB(A))	0.30	0.48	0.32	0.32	0.36	0.28	0.57	0.99

*All the correlations are at 0.001 significance level

Table S2. Association of noise with MCI[a], OR (95% CI)

	**L_{DEN} continuous (per IQR=14.2 dB(A))**	**L_{NIGHT} continuous (per IQR=13.6 dB(A))**	**L_{DEN} (threshold 65 dB(A), per 10 dB(A))**	**L_{NIGHT} (threshold 50 dB(A), per 10 dB(A))**
Overall MCI	1.10 (0.94, 1.28)	1.09 (0.94,1.27)	1.89 (1.10, 3.25)	1.35 (1.00, 1.82)
Amnestic MCI	1.15 (0.94, 1.40)	1.13 (0.93, 1.38)	2.39 (1.28, 4.45)	1.47 (1.02, 2.12)
Non-amnestic MCI	1.06 (0.87, 1.30)	1.06 (0.87, 1.30)	1.34 (0.60, 2.98)	1.22 (0.81, 1.85)

[a]Adjusted for age, sex, SES, alcohol consumption, smoking status, self-reported ETS, any regular physical activity, BMI

Table S3. Association between L_{DEN} (categorical) and overall MCI[a]

**L_{DEN}, dB(A)**	**OR (95% CI)**
< 45	Reference
45 - 55	0.98 (0.73,1.32)
55 - 65	1.08 (0.78,1.49)
65 - 75	1.21 (0.84,1.74)
> 75	1.77 (0.61,5.07)

[a]Adjusted for age, sex, SES, alcohol consumption, smoking status, self-reported ETS, any regular physical activity, BMI

A.

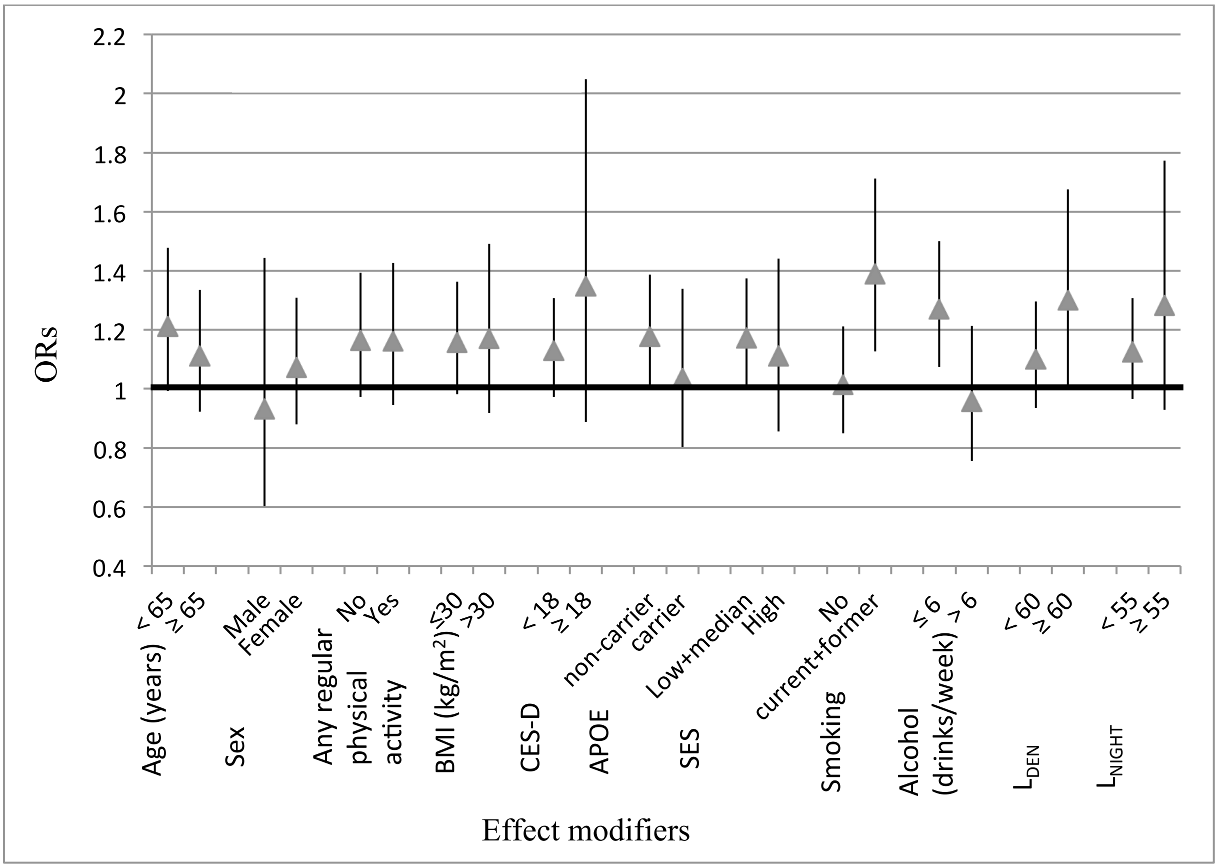
2.2
2
1.8
1.6
1.4
1.2
1
0.8
0.6
0.4
ORs
< 65
≥ 65
Male
Female
No
Yes
≤30
>30
< 18
≥ 18
non-carrier
carrier
Low+median
High
No
current+former
≤ 6
> 6
< 60
≥ 60
< 55
≥ 55
Age (years)
Sex
Any regular physical activity
BMI (kg/m2)
CES-D
APOE
SES
Smoking
Alcohol (drinks/week)
LDEN
LNIGHT
Effect modifiers

B.

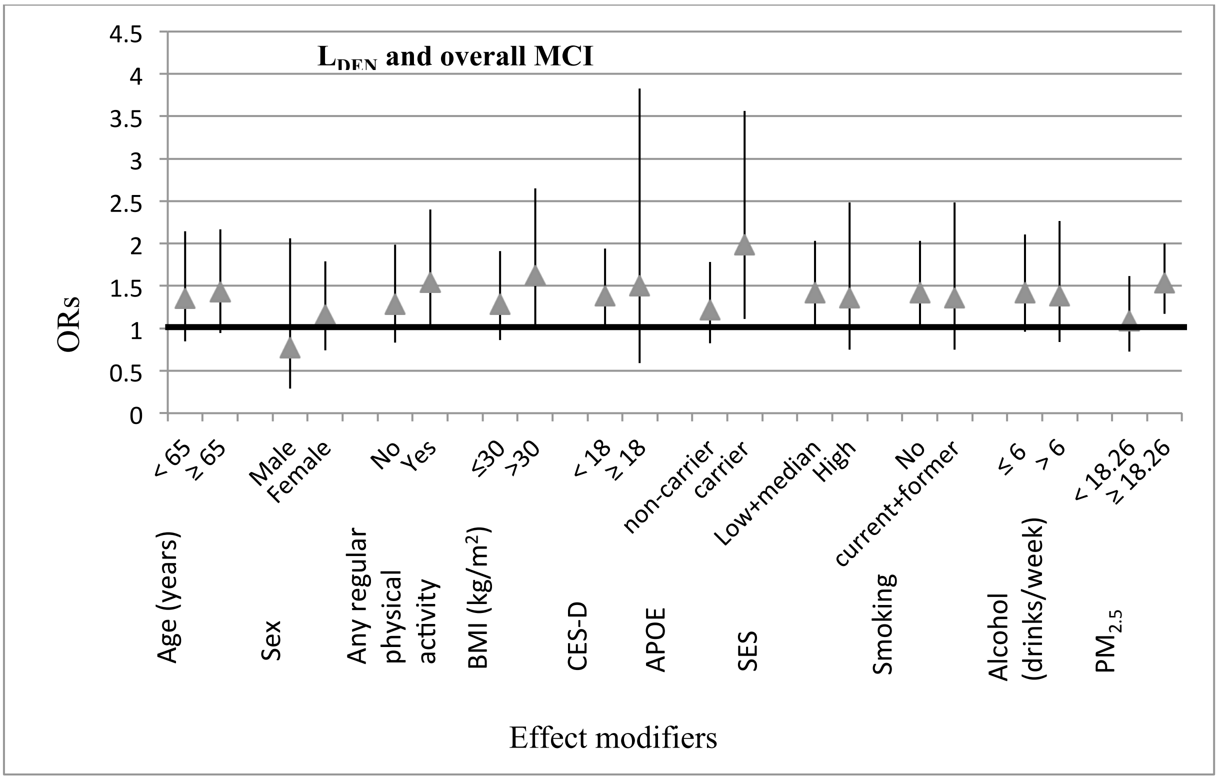
LDEN and overall MCI
4.5
4
3.5
3
2.5
2
1.5
1
0.5
0
ORs
< 65
≥ 65
Male
Female
No
Yes
≤30
>30
< 18
≥ 18
non-carrier
carrier
Low+median
High
No
current+former
≤ 6
> 6
< 18.26
≥ 18.26
Age (years)
Sex
Any regular physical activity
BMI (kg/m2)
CES-D
APOE
SES
Smoking
Alcohol (drinks/week)
PM2.5
Effect modifiers

Figure S1. Effect modification of association between exposures and overall MCI. Panel A: Associations of $PM_{2.5}$ (per IQR) with overall MCI. Panel B: Associations of L_{DEN} (per 10 dB(A)) with overall MCI. Main model adjusted for age, sex, SES, alcohol consumption, smoking status, self-reported ETS, any regular physical activity and BMI.
